# Evaluating the availability, effectiveness, and impact of primary trauma care training in Sub-Saharan Africa: A comprehensive review

**DOI:** 10.1016/j.afjem.2025.03.003

**Published:** 2025-04-15

**Authors:** Cherinet Osebo, Tarek Razek, Victoria Munthali, Respicious Boniface

**Affiliations:** a Hargelle Hospital, Emergency Surgery and Obstetrics Unit, Hargelle, Ethiopia; b School of Population and Global Health, McGill University, Canada; c Muhimbili Orthopedic Institute, Tanzania; d Muhimbili University of Health and Allied Science, Tanzania

**Keywords:** Trauma training, Sub-Saharan Africa, Primary trauma care, Trauma services improvements

## Abstract

**Background:**

Trauma is a leading cause of morbidity and mortality in sub-Saharan Africa (SSA), contributing to over 90 % of global injury-related deaths. Limited healthcare infrastructure, insufficient access to skilled healthcare professionals, and resource constraints exacerbate the trauma burden. While Advanced Trauma Life Support (ATLS) has demonstrated effectiveness in high-income settings, its high cost and limited adaptability make it less feasible in SSA. The Primary Trauma Care (PTC) course offers a cost-effective and context-appropriate alternative to ATLS. However, its implementation and impact in SSA remain underexplored. This review evaluates the availability, effectiveness, impact, and affordability of PTC courses in SSA.

**Methods:**

A comprehensive literature review was conducted across Medline, PubMed, Embase, and African Journals Online to identify studies on PTC training in SSA. Studies examining PTC's impact on injury management, knowledge, skills, patient outcomes, and cost-effectiveness were included. Inclusion criteria focused on studies conducted between 2000 and 2024 in SSA, with a focus on PTC implementation.

**Results:**

Four published PTC training studies were identified in both urban and rural settings across SSA, highlighting significant underutilization due to limited funding, infrastructure challenges, and insufficient institutional support. Among 2758 trainees, the majority were nurses (1625, 58.9 %) and clinical officers (1624, 58.8 %), followed by physicians (979, 35.5 %) and medical students (155, 5.6 %). Three studies demonstrated significant improvements in participants' knowledge, confidence, and skills. One study reported a reduction in trauma-related mortality rates. However, only one study directly assessed patient outcomes, indicating a gap in evaluating long-term impacts.

**Conclusion:**

To the best of our knowledge, this is the first comprehensive review of PTC training in SSA, offering novel insights into its effectiveness and addressing critical gaps in trauma care research for resource-limited settings. The findings demonstrate that PTC significantly enhances trauma care knowledge, confidence, and departmental capacity, making it a cost-effective and scalable training program for resource-constrained settings. However, the limited scope and frequency of PTC courses call for policymakers to prioritize expanding access to these programs and integrating them into national trauma care strategies. Collaborative efforts are needed to secure funding, increase institutional support, and conduct more institution-based studies to evaluate the long-term impact of PTC on patient outcomes, morbidity, and quality of life in SSA.

**African relevance**

• Trauma causes more deaths in SSA than major infectious diseases, exacerbated by limited healthcare infrastructure and insufficient trauma care training.
• To address these gaps, several trauma care training programs have been introduced, including ATLS, but resource constraints and cost-effectiveness limit access, particularly in SSA.
• This study investigates PTC training as an alternative to ATLS exclusively in SSA, focusing on availability, effectiveness, and addressing regional disparities in trauma care education.
• Findings will provide insights for policy development and resource allocation to improve trauma burden and outcomes in SSA.

## Introduction

Trauma and injury cause more deaths than tuberculosis, HIV, malaria, and COVID-19 combined, disproportionately affecting Sub-Saharan Africa (SSA), which accounts for over 90 % of global injury-related deaths [1,2]. This disparity results from limited infrastructure, inadequate trauma centers, resource shortages, and insufficiently trained healthcare personnel [3,4]. Trauma in SSA, including conflict, interpersonal violence, and traffic accidents, leads to significant morbidity and mortality, contributing to a global economic burden of 6 % of years lived with disability [5].

Efforts to improve trauma care in SSA include developing prehospital systems, optimizing trauma facilities, and enhancing training programs [6]. While structured training programs such as Advanced Trauma Life Support (ATLS) have proven effective in high-income countries (HICs), their implementation in SSA is hindered by high costs, limited accessibility, and lack of contextualization for low-resource settings [[7], [8], [9], [10], [11], [12]].For example, the annual cost of the ATLS program in Mongolia exceeds $10,709, with an additional $84,875 required to initially train local instructors [13]. Moreover, ATLS primarily targets physicians, excluding many frontline providers critical to trauma care in SSA. In SSA, task-shifting, where non-physician clinicians play a central role in trauma care, is a vital strategy[[13], [14], [15]].

The Primary Trauma Care (PTC) course was developed as a cost-effective, multidisciplinary, and sustainable alternative to ATLS, tailored for low-resource settings [16]. Implemented in over 70 countries (www.primarytraumacare.org), it follows a five-day cascading model: a two-day provider course, a one-day instructor course, and a subsequent provider course led by new instructors. The program emphasizes free training, online resources, and adaptable curricula. Like ATLS [10,13], PTC uses lectures, small group work, and hands-on skill sessions, covering primary and secondary surveys, airway management, and trauma care for the head, chest, abdomen, pelvis, spine, and limbs, specifically designed for low-resource settings, incorporating locally available resources[16].

However, unlike ATLS, which primarily targets physicians [13,14], PTC includes multidisciplinary providers—a key advantage in settings where non-physician clinicians play a central role in trauma care [[16], [17], [18]]. Although direct comparisons with ATLS are limited, a systematic review reports significant post-training improvements in provider knowledge, confidence, skills, and patient outcomes after PTC training [19], demonstrating critical impacts on low-resource settings. Its proven impact and widespread adoption make it a viable alternative where ATLS is impractical.

While existing trauma education studies often generalize findings across low- and middle-income countries (LMICs) [7,11,20], they fail to account for significant disparities within this group. For example, comparing China (an upper-middle-income country) with Burundi (a low-income country in SSA) highlights stark differences in resources [21], healthcare infrastructure, and the feasibility and accessibility of training programs. Such generalizations obscure the unique challenges faced in SSA, where resource constraints are more severe. Despite the growing burden of trauma in SSA, the effectiveness, cost-efficiency, and multidisciplinary approach of the PTC program have yet to be comprehensively evaluated in the region [17,19,20,22]. This gap highlights the urgent need for a focused review to assess the impact and availability of PTC training in SSA.

This review seeks to address this gap by evaluating published experiences of PTC courses in SSA, offering insights into their effectiveness, affordability, availability, and potential as a viable alternative to ATLS in the region. By analyzing PTC's impact on patient outcomes and healthcare providers' skills, knowledge, and confidence, this research will determine its suitability for multidisciplinary contexts in SSA. As the first comprehensive review focused exclusively on SSA, this study provides critical insights to inform trauma care strategies tailored to the region's unique needs.

## Materials and methods

This review followed the PRISMA–ScR guidelines: Checklist and Explanation, to ensure systematic and transparent reporting [23]. This framework was chosen to enhance the review's comprehensiveness and replicability. A comprehensive literature review was conducted to synthesize and summarize findings across multiple studies. This approach was particularly suitable for assessing PTC training in SSA, given the limited comprehensive analyses available on the topic. To ensure systematic data collection and analysis, structured research questionnaires were employed. These tools facilitated the evaluation of the availability and impact of PTC training on injury management, healthcare provider competencies, and patient outcomes in SSA.

## Search strategy

A comprehensive literature search was conducted to identify studies related to the PTC course in SSA. A trained research librarian assisted in refining the search strategy and provided expert advice on database selection and search terms. The following databases were searched: MEDLINE (Ovid interface), PUBMED (Ovid interface), Embase (Ovid interface), African Journals Online, and Global Health (Ovid interface). The search terms used were:

"Primary Trauma Care*" OR "Trauma Course*" OR "Injury Care Education*" OR "PTC training*" AND “Sub-Saharan Africa*” OR “Africa*”

The search was restricted to peer-reviewed articles published between January 1st, 2000, and October 31st, 2024, and written in English. To ensure thoroughness, the reference lists of relevant publications were also manually searched for additional studies related to trauma training.

## Inclusion and exclusion criteria

Studies were included if they focused on PTC training in SSA and explored its impact on injury management. Participants were limited to facility-based healthcare practitioners (e.g., doctors, nursing staff, clinical officers, residents, and medical students). Key inclusion criteria included studies that assessed knowledge, skills, and confidence improvements of participants, or patient outcomes, such as mortality, morbidity, or complications.

Studies were excluded if the training focused on prehospital care providers or community health workers; the courses lacked information on the structure of the education provided; or studies used only subjective measurement tools without evaluating skills or including non-English papers.

## Study selection and data extraction

The study selection process was conducted by CO and TR, who independently screened the titles and abstracts of the identified publications to assess relevance. In cases of disagreement, the reviewers discussed the articles and reached a consensus. After the initial screening, the full texts of selected studies were reviewed, and exclusion criteria were applied. Any duplicate studies identified were removed through a systematic review of the database results. Data were extracted systematically using Excel, focusing on: training locations and course descriptions; certified healthcare providers involved; outcomes related to participant improvements in skills and patient outcomes; and financial considerations (cost-effectiveness), where available.

## Framework for data analysis

We adapted Kirkpatrick's Four-Level Training Evaluation Model to assess the effectiveness of the PTC training programs. The model evaluates four levels: Reaction, Learning, Behavior, and Results, each with specific outcome measures (Table 1) [24]. We selected Kirkpatrick's model because of its widespread use in evaluating healthcare training programs, offering a comprehensive framework that spans participant reactions to patient outcomes. To ensure its relevance to the unique challenges of SSA, we adapted the model by considering factors such as resource limitations, the diversity of healthcare providers, and the varying levels of trauma care infrastructure across the region.

**Table 1:** Kirkpatrick model for evaluating training.

Kirkpatrick Level	Description	Training outcome measures
Level 1 reaction	Participants’ perceptions of the training's value and relevance to their daily duties.	Subjective evaluations
Level 2 learning	Participants acquire the expected knowledge, skills, confidence, and commitment from the training.	Objective pre/post-tests Confidence measurements
Level 3 behavior	Participants apply the objectives and skills learned during training to their daily tasks.	Objective skill measurement using simulation cases, OSCE, or assessments of actual injured patients.
Level 4 results	The training influences participants’ performance and outcomes.	Improvements in trauma care, including reductions in mortality, morbidity, complications, or overall trauma system efficacy.

## Data synthesis

Data from the selected studies were synthesized qualitatively, with a focus on identifying recurring themes related to training effectiveness, participant skill development, and impact on patient outcomes. Additionally, findings were compared across different SSA countries to detect patterns and disparities in the effectiveness of PTC training, considering the region's diverse healthcare contexts. The synthesis was carried out by VM and RB, who independently reviewed the data and discussed discrepancies to reach a consensus.

## Quality assurance

To ensure transparency, the studies selected were assessed for quality based on their methodological rigor, with particular attention to the reporting of training outcomes and relevant metrics. The quality assessment was carried out by VM and RB, using a standardized checklist to score studies on key criteria such as the clarity of training structures, the use of objective outcome measures, and the reporting of detailed results. Studies that relied solely on subjective measures or lacked detailed documentation of training structures were excluded.

## Results

**Fig. 1:**
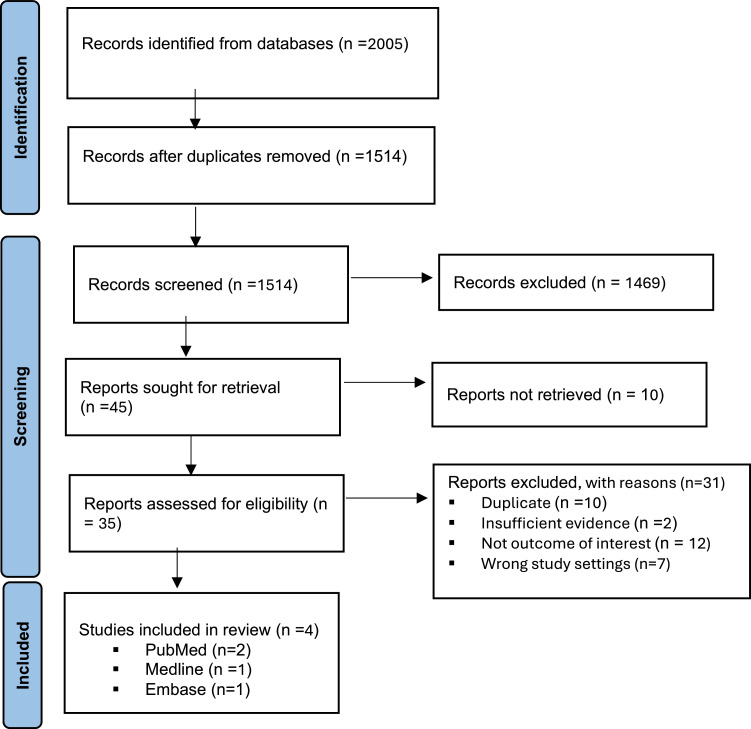
Flow diagram chart for literature review process.

Fig. 1 illustrates the process of identifying and selecting the articles included in this review. A total of 2005 citations were identified through searches in Medline, PUBMED, Embase, Global Health, and African Journals Online. After removing duplicates and screening titles and abstracts, 1514 records were reviewed, leading to 35 articles undergoing full-text screening. Of these, 4 articles met the inclusion criteria and were included in the review [[25], [26], [27], [28]]. All selected articles were original research and peer-reviewed. These 4 studies were chosen for their relevance to the impact of PTC training, specifically in SSA. Our review highlights the limited scope of research on this topic in the region.

## Audience and course settings

A total of 2758 participants attended the PTC courses across all studies included. The training took place in both urban and rural settings within SSA. The distribution of participants is as follows: 1625/2758 (58.9 %) nurses, 1624/2758 (58.88 %) clinical officers, 979/2758 (35.5 %) physicians, and 155/2758 (5.6 %) medical students (Table 2).

**Table 2:** Primary trauma care (PTC) course description across sub-Saharan Africa.

Author (Year)	Location	Course	Attendees	Assessments Modes	Key Outcomes	Kirkpatrick Levels
Peter (2015)	COSECSA Countries	PTC	540 Physicians; 260 Nurses; 119 Clinical officers, and 111 Medical Students	30 pre-and post-MCQ exams, and 8 confidence matrix items	■ Mean knowledge increased from 58 % to 77 % (p < 0.05). ■ Confidence improved from 68 % to 90 % (mean gain of 22 % , p < 0.05)	2
Nogaro (2015)	*COSECSA countries	PTC	240 doctors/surgeons and 105 non-doctors	Thirty pre- and post-MCQ exams alongside 8 confidence matrix items	■ Median knowledge improved from 70 % to 87 % (*p* < 0.05). ■ 91 % demonstrated a significant knowledge gain. ■ Confidence in trauma management improved by 20 %.	2
Ologunde (2017)	⁎⁎⁎COSECSA countries	PTC	253 Physicians; 98 Nurses; 44 Medical students and 40 Clinical officers	A survey following training; immediately and six months after the course completion	■ 92.7 % reported improved trauma management skills six months post-training. ■ 52.8 % adopted the ABCDE approach. ■ Departmental changes were moderate (30 %), with 23 % reporting no change. ■ Only 24.8 % perceived improvements in trauma patient mortality and morbidity (*p* < 0.01).	4
Tolppa, 2020	Democratic Republic of the Congo	PTC	23 nurses and 36 doctors	20 pre- and post-MCQ exams alongside 8 confidence matrix items, and repeated within two years	■ Sustained knowledge gains were observed over two years. ■ Although confidence levels declined significantly (*p* = 0.03). ■ 95 % of participants emphasized the importance of trauma services. ■ 52 attendees highlighted the need for alternative procedures to manage local patients effectively. ■ 36 respondents noted significant equipment shortages impacting trauma care.	2

⁎ COSECSA (College of Surgeons of East, Central, and Southern Africa) member countries: Kenya, Malawi, Uganda, Ethiopia, Rwanda, Mozambique, and Zimbabwe.

⁎⁎⁎ COSECSA member countries: Burundi, Ethiopia, Kenya, Malawi, Mozambique, Rwanda, Tanzania, Uganda, Zambia, and Zimbabwe.

The courses were delivered in both urban and rural settings, with urban areas typically offering better access to resources, while rural settings presented unique challenges such as limited infrastructure and fewer trained healthcare professionals [26]. The course settings might have influenced the outcomes, as participants from rural areas could have faced different constraints in applying their newly acquired knowledge and skills. However, no direct comparisons were made between urban and rural outcomes.

Except for one study conducted at a single site [26], other PTC courses were delivered across the ten member countries of the College of Surgeons of East, Central, and Southern Africa (COSECSA) [25,27,28]. The COSECSA-Oxford-Orthopedic Link initiative, funded by the United Kingdom, coordinated the PTC programs in collaboration with the University of Oxford. The program followed a 2:1:2 cascade model, with an initial 2-day provider training, a 1-day instructor course, and a 2-day provider course led by newly certified instructors. Overseas instructors from developed countries taught the initial 2-day provider course, despite the program being coordinated by COSECSA. This international involvement may have enhanced the credibility and expertise of the program, although future studies should assess the sustainability and local ownership of these initiatives.

## Local adaptability and engagement

All studies involved local stakeholders in co-facilitating, delivering, and organizing the courses [[25], [26], [27], [28]]. Prior needs assessments were conducted to identify local challenges and resources, including workforce capacity, existing training programs, infrastructure needs, and the burden of trauma and acute surgical diseases. These assessments also highlighted financial constraints and informed decisions regarding the program's adaptability and delivery mode. These local assessments were crucial for tailoring the training to the specific contexts of different SSA regions. In some settings, financial constraints led to adjustments in course delivery, such as offering more affordable materials or shorter training periods. Local stakeholders’ involvement in the facilitation process ensured that the training was culturally and contextually appropriate, which may have contributed to higher engagement and better learning outcomes.

## Course effectiveness

### Participant-related outcomes (Knowledge, skill, and confidence)

**Knowledge Improvement:** Three articles reported significant increases in participants' knowledge following the PTC training, as measured by multiple-choice question (MCQ) tests [25,26^.^^28^]. In two studies, participants demonstrated a statistically significant improvement in trauma care knowledge (*p* < 0.05), with knowledge gains ranging from 17 % to 67.5 % [25,28]. In the third study, participants showed sustained knowledge retention, with improvements maintained 24 months post-course [26]. These findings underscore the effectiveness of PTC training in enhancing trauma care knowledge, which is crucial in SSA's resource-limited settings.

**Confidence**: Two studies assessed participants' confidence in handling trauma cases after completing the PTC course [25,28]. Both studies reported significant improvements: 22 % and 20 % increases in confidence, respectively, which were statistically significant (*p* < 0.05). Non-physician participants (nurses and clinical officers) exhibited slightly higher confidence gains compared to physicians (20 % vs. 17 % and 22 % vs. 18 %) [25,28]. These findings are noteworthy as they suggest that non-physician healthcare workers—who are often the first point of contact in trauma care in SSA—may benefit more in terms of confidence from this training due to its practical focus and the direct application of skills to their daily responsibilities.

**Skill Improvement**: Ologunde et al. conducted an immediate post-training assessment and found that 13.1 % of participants improved specific skills in trauma care [27]. A follow-up survey six months later indicated that 53 % of participants adopted an ABCDE (Airway, Breathing, Circulation, Disability, Exposure) approach to trauma care, and 92.7 % reported improved trauma management within their departments. Participants also noted improvements in teamwork, communication, and pre-hospital care. These results suggest that while immediate skill improvement may be modest, the long-term impact on clinical practice and team dynamics is significant.

**Long-Term Knowledge Retention**: In the study by Tolppa et al., 26 immediate post-course assessments showed improved knowledge and confidence (*p* < 0.01). However, at the 24-month follow-up, while knowledge retention remained high, confidence decreased (*p* = 0.03). Many participants reported a lack of operative infrastructure and expressed concerns about managing trauma cases with limited resources. Nonetheless, most agreed that the course contributed positively to trauma care management in their facilities. These results highlight the challenges in maintaining confidence and applying trauma care knowledge in the face of infrastructure limitations, suggesting the need for follow-up training or supportive measures in resource-limited settings.

### Patient-Related outcomes (Mortality, morbidity, and complications)

Except for Ologunde et al., [27] none of the studies assessed patient outcomes post-PTC training. Ologunde et al. reported that 25 % of participants observed reductions in mortality and morbidity within their departments six months after completing the course (*p* < 0.001). However, these results were not systematically compared with baseline measures, and only a few respondents provided evidence of patient outcomes. The lack of consistent patient outcome data is a significant gap in the literature. Future research should prioritize a systematic collection of patient outcomes to assess the true impact of PTC training on trauma care quality and patient survival.

## Cost-Effectiveness

Only one study evaluated the cost-effectiveness of PTC training. Peter et al. reported that the cost of a primary 5-day PTC course was approximately $7706, while the cost for a cascading course was $5533 [25]. The cascading model was found to be more cost-effective, with the per-participant cost being $184 for the cascade model compared to $256 for the primary course. These findings are important for scaling up trauma care training programs in SSA settings, as the cascade model's lower cost makes it a more viable option for widespread implementation. Further studies should evaluate the long-term cost-effectiveness of these models, considering not only direct costs but also the potential savings associated with improved trauma care and reduced morbidity and mortality.

## Discussion

This review highlights a significant gap in the availability of PTC training within SSA, emphasizing the urgent need to expand these courses or develop alternative trauma care training programs. Designed to address such challenges, the PTC course offers an affordable and scalable solution through its 'cascading model,' which trains local instructors and providers, enabling sustainable knowledge transfer and broader access, particularly in remote areas [[16], [17], [18], [19]].

Despite these strengths, our review identified only four studies assessing the PTC course's impact in SSA, underscoring its limited availability. Barriers such as financial constraints, logistical challenges, and geographic limitations hinder broader implementation. Rural regions often lack access to centralized training, and the success of the cascading model depends on the availability of skilled local instructors—a significant challenge in underserved areas. To ensure the sustainability and scalability of PTC programs, ongoing support, including refresher courses and instructor training, is crucial [[25], [26], [27], [28]]. These findings underscore the need for targeted strategies to improve the availability and sustainability of PTC training in SSA, ensuring equitable access to trauma care education in resource-constrained settings.

The studies reviewed demonstrate that PTC courses significantly enhance participants' knowledge and confidence in trauma management. Similar to findings in other regions [20,29], these improvements were particularly notable among non-physician healthcare providers, such as nurses and clinical officers, emphasizing the critical role of task-shifting in addressing the shortage of skilled healthcare providers in SSA[25,28]. These findings align with the study's aim of evaluating the impact of PTC training and highlighting its importance in strengthening trauma care systems in resource-limited settings. Furthermore, the enhanced knowledge and confidence among participants contributed to tangible improvements in patient care, including better decision-making, faster response times, and more effective trauma management, thereby improving overall healthcare efficiency.

PTC training has contributed to measurable improvements in trauma care facilities and infrastructure. Participants in the reviewed studies reported advancements such as faster trauma care delivery and reduced pre-hospital transfer times [27], consistent with findings from other studies [[20], [30]]. These outcomes align with the study's focus on the broader impact of PTC courses in SSA, particularly their role in strengthening healthcare systems’ capacity to manage trauma cases more effectively. By integrating PTC into existing healthcare frameworks, such as the World Health Organization's (WHO) Emergency Care Systems framework, these courses enhance facility capacity and resource utilization [31]. Additionally, the cascading training model, which trains local instructors, ensures the sustainability of these improvements at the institutional level. This approach is vital for building resilient trauma care systems in SSA, where healthcare infrastructure often faces significant challenges.

Although PTC training improves knowledge and skills, its direct impact on patient outcomes, such as mortality and morbidity, remains unclear [18,27]. While some studies report better outcomes in facilities using PTC principles [27], the lack of control groups and confounding factors limits these findings. Unlike ATLS [[8], [9], [10]], which demonstrates clear mortality reductions in HICs, PTC lacks robust longitudinal evidence in SSA [[26], [27], [28]]. Future research should prioritize controlled, long-term studies to clarify its effect on survival and recovery rates.

A key concern highlighted in this review is the sustainability of the knowledge gained through PTC training. Although studies show that knowledge is retained up to 24 months after training [26], there is a noticeable decline in both confidence and retention over time, like other trauma care courses like ATLS [33]. Without periodic recertification or refresher courses, the benefits of PTC training may diminish, ultimately affecting the quality of care provided. Given the limited resources for regular training updates in SSA, it is crucial to integrate recertification mechanisms into the PTC framework to ensure sustained knowledge and skill retention. WHO guidelines emphasize the need for ongoing education to maintain the effectiveness of trauma care [20,32,33]. Implementing such mechanisms within the PTC framework would maximize its long-term impact and help ensure that healthcare providers in SSA maintain high standards of care.

Cost-effectiveness analyses for PTC training in SSA are limited, with only one study addressing the financial aspects of the program [25]. While the primary costs are significant, the cascading model offers a more cost-effective approach, especially considering its scalability and the widespread dissemination of knowledge. Despite being more affordable than programs like ATLS [33], the sustainability of PTC training remains a concern, as funding is often dependent on external sponsors [17]. Like other trauma courses [20], the long-term viability of PTC training requires securing sustainable funding models that prioritize building local capacity and reducing reliance on external support. Further research on the cost-effectiveness of PTC in SSA is also needed to assess its long-term financial sustainability and broader impact on healthcare systems.

## Limitations

This review has several limitations that should be considered. The studies included in this review varied in terms of methodology, outcome measures, and program implementations, which introduces variability in the results. Some studies relied on subjective outcome measures, which can introduce bias and affect the consistency and reliability of the findings. Additionally, the limited number of studies available on the PTC program in SSA means that the findings may not be fully representative of all contexts within the region. These limitations highlight the need for more rigorous studies with consistent methodologies, objective outcome measures, and a larger sample size to better capture the nuances of PTC training in SSA.

## Conclusions

This review provides a detailed analysis of PTC training programs in SSA, addressing a critical gap in the literature regarding trauma care education in the region. The findings demonstrate that PTC courses present a promising and cost-effective alternative to more expensive programs like ATLS, resulting in significant improvements in trauma care knowledge, skills, and confidence among healthcare providers. PTC training has also contributed to improvements in trauma care infrastructure and systems at the institutional level. Despite these benefits, the scalability of PTC training remains limited in SSA, and there is a lack of long-term studies linking PTC training to patient outcomes. Furthermore, challenges related to knowledge retention and sustainability highlight the need for future research to explore ways to enhance the long-term impact of PTC programs.

## Recommendations

To maximize the impact of PTC training in SSA, it is essential to expand training programs, implement recertification processes, and conduct further research on the long-term effects of PTC on patient outcomes. Additionally, securing sustainable funding and building local capacity will be crucial for the program's continued success and widespread adoption. These steps will help ensure that PTC training continues to enhance trauma care outcomes across SSA.

## Dissemination of results

Results from this narrative review were shared with the research community and stakeholders through academic presentations at local conferences. Additionally, the findings were disseminated via peer-reviewed journal publications and shared with relevant healthcare providers through professional networks and newsletters to facilitate broader awareness of the findings within the context of trauma care in Sub-Saharan Africa

## Authors' contributions

CO contributed 85 %; TR, VM, and RB contributed 5 % each. All authors approved the version to be published and agreed to be accountable for all aspects of the work.

## Declaration of competing interest

The authors declare that they have no known competing financial interests or personal relationships that could have appeared to influence the work reported in this paper.

## References

[1] Murray C.J., Ortblad K.F., Guinovart C., Lim S.S., Wolock T.M., Roberts D.A. (2013). Global, regional, and national incidence and mortality for HIV, tuberculosis, and malaria during 1990-2013: a systematic analysis for the Global Burden of Disease Study. Lancet.

[2] World Health Organization (2021). https://www.who.int/news-room/fact-sheets/detail/injuries-and-violence.

[3] Mock C., Joshipura M., Arreola-Risa C., Quansah R. (2012). An estimate of the number of lives that could be saved through improvements in trauma care globally. World J Surg.

[4] Hanche-Olsen T.P., Alemu L., Viste A., Wisborg T., Hansen K.S. (2015). Evaluation of training program for surgical trauma teams in Botswana. World J Surg.

[5] Bundu I., Lowsby R., Vandy H.P., Kamara S.P., Jalloh A.M., Scott C.O.S. (2019). The burden of trauma presenting to the government referral hospital in Freetown, Sierra Leone: an observational study. Afr J Emerg Med.

[6] Reynolds T.A., Stewart B., Drewett I., Salerno S., Sawe H.R., Toroyan T. (2017). The impact of trauma care systems in low- and middle-income countries. Annu Rev Public Health.

[7] Mock C.N., Quansah R., Addae-Mensah L., Donkor P. (2005). The development of continuing education for trauma care in an African nation. Injury.

[8] Carmont M.R. (2005). The advanced trauma life support course: a history of its development and review of related literature. Postgrad Med J.

[9] Mohammad A., Branicki F., Abu-Zidan F.M. (2014). Educational and clinical impact of advanced trauma life support (ATLS) courses: a systematic review. World J Surg.

[10] Jayaraman S., Sethi D. (2009). Advanced trauma life support training for hospital staff. Cochrane Database Syst Rev.

[11] Petroze R.T., Byiringiro J.C., Ntakiyiruta G., Briggs S.M., Deckelbaum D.L., Razek T. (2015). Can focused trauma education initiatives reduce mortality or improve resource utilization in a low-resource setting?. World J Surg.

[12] Chokotho L., Jacobsen K.H., Burgess D., Labib M., Le G., Peter N. (2016). A review of existing trauma and musculoskeletal impairment (TMSI) care capacity in East, Central, and Southern Africa. Injury.

[13] Kornfeld J.E., Katz M.G., Cardinal J.R., Bat-Erdene B., Jargalsaikhan G., Nunez J. (2019). Cost analysis of the Mongolian ATLS(c) program: a framework for low- and middle-income countries. World J Surg.

[14] American College of Surgeons (2022). https://www.facs.org/-/media/files/qualityprograms/trauma/atls/intnlpromulgation.ashx.

[15] Hammerstedt H., Maling S., Kasyaba R., Dreifuss B., Chamberlain S., Nelson S. (2014). Addressing world health assembly resolution 60.22: a pilot project to create access to acute care services in Uganda. Ann Emerg Med.

[16] Wilkinson D., McDougall R. Primary trauma care. Anesthesia. 2007;62 Suppl 1:61–4: 10.1111/j.1365-2044.2007.05301.x. 17937716

[17] Jawaid M., Memon A.A., Masood Z., Alam S.N. (2013). Effectiveness of the primary trauma care course: is the outcome satisfactory?. Pak J Med Sci.

[18] Ley Greaves R.A., Wilkinson L.F., Wilkinson D.A (2017). Primary trauma care: a 20-year review. Trop Doct.

[19] Kadhum M., Sinclair P., Lavy C. (2020). Are primary trauma care (PTC) courses beneficial in low- and middle-income countries - a systematic review. Injury.

[20] Osebo C., Razek T., Deckelbaum D., Grushka J., Khwaja K., Fazlollahi A. (2024). Enhancing trauma care through innovative trauma and disaster team response training: a blended learning approach in Tanzania. World J Surg.

[21] UN (2022). https://hdr.undp.org/data-center/human-development-index#/indicies/HDI.

[22] Osebo C., Razek T., Grushka J., Deckelbaum D., Khwaja K., Munthali V., Boniface R. (2024). Impacting trauma care in resource-limited settings: lessons learned from Tanzania's web-based trauma registry initiatives. World J Surg.

[23] Tricco A.C., Lillie E., Zarin W., O'Brien K.K., Colquhoun H., Levac D. (2018). PRISMA extension for scoping reviews (PRISMA-ScR): checklist and explanation. Ann Intern Med.

[24] Kirkpatrick D.L. (1975).

[25] Peter N.A., Pandit H., Le G., Nduhiu M., Moro E., Lavy C. (2016). Delivering a sustainable trauma management training program tailored for low-resource settings in East, Central and Southern African countries using a cascading course model. Injury.

[26] Tolppa T., Vangu A.M., Balu H.C., Matondo P., Tissingh E. (2020). Impact of the primary trauma care course in the Kongo Central province of the Democratic Republic of Congo over two years. Injury.

[27] Ologunde R., Le G., Turner J., Pandit H., Peter N., Maurer D. (2017). Do trauma courses change practice? A qualitative review of 20 courses in East, Central and Southern Africa. Injury.

[28] Nogaro M.C., Pandit H., Peter N., Le G., Oloruntoba D., Muguti G. (2015). How useful are Primary Trauma Care courses in sub-Saharan Africa?. Injury.

[29] Cervero R.M., Gaines J.K. (2015). The impact of CME on physician performance and patient health outcomes: an updated synthesis of systematic reviews. J Contin Educ Health Prof.

[30] Qin L.J., Shi X.-P., Chang Y.-X., Li F.-L., Wang P. (2019). Systemic analysis of pre-hospital trauma emergency treatment in Zhengzhou. J Acute Dis.

[31] World Health Organization (2017). https://www.who.int/emergencycare/emergencycare_infographic/en/.

[32] Mock C. (2009).

[33] Radvinsky D.S., Yoon R.S., Schmitt P.J., Prestigiacomo C.J., Swan K.G., Liporace F.A. (2012). Evolution and development of the advanced trauma life support (ATLS) protocol: a historical perspective. Orthopedics.

